# Practical Rules for the Suppression of Arterial Hemorrhage

**Published:** 1852-03

**Authors:** 


*Practical Rules for the Suppression of Arterial Hemorrhage.* By Professor Syme.—In the first place, you should hold it established, that it is always desirable, if possible, to arrest bleeding from arteries by means applied at the seat of injury. Secondly, you may be assured that bleeding at and below the wrist, and at and below the ankle, is always under the control of the pressure, provided it be properly employed,—that is, not superficially, but from lint, or some other suitable substance being introduced into the wound, and made to press directly upon the orifice of the vessel. Thirdly, in wounds of all arteries, accessible between the limits just mentioned and the heart, the vessel should be exposed at the seat of injury, and tied on both sides of the wound it has sustained. This principle has been so loudly maintained by Mr. Guthrie, that I believe some people have given him the credit of its origin; but it has been long established as a sound principle of practice by surgeons of the highest eminence both at home and abroad, and more especially by Mr. John Bell, of Edinburgh, in whose 'Principles of Surgery' you will find many graphic and impressive lessons of the effects resulting from inattention to it, and also from its regard.

One evening I received a message from the Northern Railway, that there was a steamboat waiting at Granton to carry me across the Frith to Burntisland, where a special train would he ready to proceed onwards, but whither, or for what purpose, there was no information. Having traveled a considerable distance, I met several practitioners, of great experience and intelligence, who were suffering much anxiety in regard to a youth, in whose forearm an incision for an abscess had bled profusely. As it was quite away from the radial artery, the ulnar was concluded to be the source of hemorrhage, and had been sought for by dissection upwards towards the elbow, along the course of the muscles, between which it is wont to run, but without success; and, as the patient seemed little able to bear any further loss of blood, it was deemed desirable to have a consultation as to the most efficient measure of relief, even though it might involve ligature of the humeral artery, or removal of the limb. Acting upon the principle above mentioned, I scratched away the clot at the bleeding point, from which a copious stream instantly issued, but arresting this with my thumb, pressure being at the same time made upon the humeral, I dissected a little through the adjacent texture, and brought into view a large artery, under which a double ligature was passed, and tied on both sides of an aperture distinctly visible in its coats. In less time than I have taken to describe the process, the patient was thus transferred from a state of extreme danger to one of perfect safety. The artery was obviously the ulnar, which had come off higher than usual from the humeral, and pursued an irregular course externally to the fascia of the forearm, thus explaining how it had been wounded by the superficial incision, and how it had escaped the deep dissection.

The fourth rule I have to offer is, that when an aneurism forms after the wound of an artery, the same means should be employed as in the first instance, unless the vessel concerned be of a large size, and admits of having a ligature applied to it, without the intervention of any large branch between the seat of obstruction and the wound. The formerly not uncommon case of aneurism at the bend of the arm, as a consequence of the humeral artery being wounded in venesection, affords a good illustration of the advantage resulting from attention to this rule, since relief was thus afforded much more easily, safely, and securely, than by ligature of the humeral further up the arm.

To illustrate the exception mentioned, I may relate the case of a young man who, in one of the most remote of the Orkney Islands, accidentally thrust the blade of a knife into the middle of his thigh, so as to wound the femoral artery. The blood gushed forth with great violence, but was restrained by a compress, formed of eight half-crowns, wrapped in a piece of cloth. The wound healed, and an aneurism soon afterwards appearing, he was sent here to my care. Respect for the general principle, and suspicion from the purring sound, that there was a communication between the artery and vein, suggested considerations which were opposed to ligature of the femoral, but I nevertheless preferred this operation, as the ligature could be applied without the intervention of any considerable branch; and I accordingly performed it, with the happiest result.

The following case will show the danger of not strictly limiting exceptions to the rule within the limits which have been mentioned. A middle aged woman, in a country town, while walking up a steep and slippery ascent, and carrying a knife, with which she had just killed a pig, fell, and thrust the sharp point of the blade completely through her leg, a little below the knee, entering between the tibia and fibula, and issuing at the lower part of the popliteal space. Blood gushed from both openings, but when she was laid in bed ceased, and did not return. At the end of a fortnight, the wounds having healed, she attempted to walk, and found that a swelling had taken place at the seat of injury, on account of which, by the advice of her medical attendant, she came here to be under my care. On examination, I found a large pulsating tumor in the forepart of the leg, immediately below the knee, and another of equal size in the popliteal cavity.

Feeling unable to determine whether the anterior or posterior tibial, or the popliteal artery itself, was the vessel wounded, and, on the whole, being inclined to think that the one last mentioned was most probably concerned, in which case ligature of the femoral would be the proper course, I adopted this measure. No bad consequences followed the operation, the tumors ceased to pulsate, and favorable expectations were entertained of the result for two or three weeks, when the anterior wound below the knee opened and bled profusely. I dilated it freely, evacuated the cavity of its fluid and coagulated contents, and applied firm pressure between the tibia and fibula, whence the blood was found to issue. Mortification followed, and I performed amputation, without saving the patient's life. There can be no doubt that, in this case, if the true state of matters could have been ascertained, and a ligature applied to the anterior tibial, which was divided just before it passed through the interosseous ligament, both the limb and life of the patient would have been preserved.—*Monthly Journal of Med. Science.*

